# Maternal Pre-Pregnancy Obesity Attenuates Response to Omega-3 Fatty Acids Supplementation During Pregnancy

**DOI:** 10.3390/nu10121908

**Published:** 2018-12-04

**Authors:** Carmen Monthé-Drèze, Annie Penfield-Cyr, Marcela C. Smid, Sarbattama Sen

**Affiliations:** 1 Department of Pediatric Newborn Medicine, Brigham and Women’s Hospital, Boston, MA 02115, USA; apenfield-cyr@bwh.harvard.edu (A.P.-C.); ssen2@bwh.harvard.edu (S.S.); 2 Division of Maternal Fetal Medicine, University of Utah Health, Salt Lake City, UT 84132, USA; marcela.smid@hsc.utah.edu; 3 School of Medicine, Harvard University, Boston, MA 02115, USA

**Keywords:** maternal obesity, pre-pregnancy BMI, omega-3 supplementation, pregnancy, PUFA, inflammation, n-6/n-3 ratio

## Abstract

Maternal obesity is associated with adverse offspring outcomes. Inflammation and deficiency of anti-inflammatory nutrients like omega(n)-3 polyunsaturated fatty acids (PUFA) may contribute to these associations. Fetal supply of n-3 PUFA is dependent on maternal levels and studies have suggested that improved offspring outcomes are associated with higher maternal intake. However, little is known about how maternal obesity affects the response to n-3 supplementation during pregnancy. We sought to determine (1) the associations of obesity with PUFA concentrations and (2) if the systemic response to n-3 supplementation differs by body mass index (BMI). This was a secondary analysis of 556 participants (46% lean, 28% obese) in the Maternal-Fetal Medicine Units Network trial of n-3 (Docosahexaenoic acid (DHA) + Eicosapentaenoic acid (EPA)) supplementation, in which participants had 2g/day of n-3 (*n* = 278) or placebo (*n* = 278) from 19 to 22 weeks until delivery. At baseline, obese women had higher plasma n-6 arachidonic acid concentrations (*β*: 0.96% total fatty acids; 95% Confidence Interval (CI): 0.13, 1.79) and n-6/n-3 ratio (*β*: 0.26 unit; 95% CI: 0.05, 0.48) compared to lean women. In the adjusted analysis, women in all BMI groups had higher n-3 concentrations following supplementation, although obese women had attenuated changes (*β* = −2.04%, CI: −3.19, −0.90, interaction *p* = 0.000) compared to lean women, resulting in a 50% difference in the effect size. Similarly, obese women also had an attenuated reduction (*β* = 0.94 units, CI: 0.40, 1.47, interaction *p* = 0.046) in the n-6/n-3 ratio (marker of inflammatory status), which was 65% lower compared to lean women. Obesity is associated with higher inflammation and with an attenuated response to n-3 supplementation in pregnancy.

## 1. Introduction

Currently over 60% of women in the United States are overweight or obese prior to pregnancy [1]. Maternal obesity is associated with increased risk of adverse maternal and infant outcomes including preterm delivery, macrosomia, and adverse child metabolic and neurodevelopmental outcomes [2,3]. The metabolic environment of an obese pregnant woman is characterized by chronic dysregulated inflammation and oxidative stress, which may play a role in the developmental programming of these adverse outcomes [4,5,6,7,8].

Obesity-related inflammation can originate from intrinsic and extrinsic (dietary) sources. Intrinsically, excessive accretion of adipose tissue and concurrent macrophage activation lead to increased expression of pro-inflammatory cytokines [4]. Furthermore, women with a higher pre-pregnancy body mass index (BMI) were found to have poorer dietary quality, characterized by a higher intake of trans and saturated fats [9]. This dietary profile may further contribute to the pro-inflammatory milieu in obese pregnancies. 

Polyunsaturated fatty acids (PUFAs) and their downstream metabolites regulate inflammation and immune cell function and are often considered markers of inflammation. There are two main families of PUFAs, the omega-6 and omega-3 families, and their metabolism has been described previously [10,11,12,13]. Omega (n)-3 PUFA (docosahexaenoic acid (DHA); eicosapentaenoic acid (EPA)) metabolism produces specialized anti-inflammatory lipid mediators (resolvins, protectins, and maresins), while eicosanoids (thromboxanes, 4-series leukotrienes, and certain 2-series prostaglandins) derived from n-6 PUFAs are generally considered more pro-inflammatory [10,11]. The major substrate for the synthesis of inflammatory eicosanoids is arachidonic acid (AA), although Serhan et al. has shown that not all AA metabolites are pro-inflammatory [12,13]. For example, AA-derived lipoxins are important anti-inflammatory proresolving mediators that are involved in the resolution of inflammation. These n-3 and n-6 PUFA mediators alter the transcriptional regulation of genes that are involved in inflammation and lipid metabolism [4,10], as well as immune regulation [11]. The optimal balance of n-6 to n-3 PUFA (n-6/n-3 PUFA ratio) has been shown to reduce obesity-related inflammation and oxidative stress [14,15,16,17,18]. During pregnancy, higher intake of n-3 PUFAs and/or fish, its primary dietary source, is associated with improved fetal growth [19,20], lower offspring adiposity and atopy [21,22,23], and improved neurodevelopmental outcomes [24,25]. 

Fetal supply of PUFAs is dependent on maternal intake and systemic concentrations during pregnancy, as the ability of both the fetus and the placenta to synthesize long chain PUFAs from essential PUFA is limited [26]. PUFAs also play a crucial role in fetal and postnatal brain development. Therefore, to optimize pregnancy outcomes and offspring health, the American College of Obstetricians and Gynecologists recommends that pregnant and lactating women consume at least two servings/week of low mercury fish and/or seafood, equivalent to 200 mg of Docosahexaenoic acid (DHA) [27]. However, little is known about whether women who enter pregnancy at a higher BMI have the same systemic response to n-3 PUFAs intake as lean women. 

The objectives of this study were to determine 1) any associations between maternal pre-pregnancy obesity and concentrations of PUFAs in mid-pregnancy and 2) whether obesity attenuates the systemic concentrations of key PUFAs in response to n-3 supplementation in pregnancy. We hypothesized that pre-pregnancy obesity would be associated with a higher baseline n-6/n-3 PUFA ratio and that n-3 PUFA supplementation would lead to an attenuated rise in systemic n-3 PUFA concentrations in obese compared to lean women.

## 2. Materials and Methods 

### 2.1. Study Design and Participants

**Figure 1:**
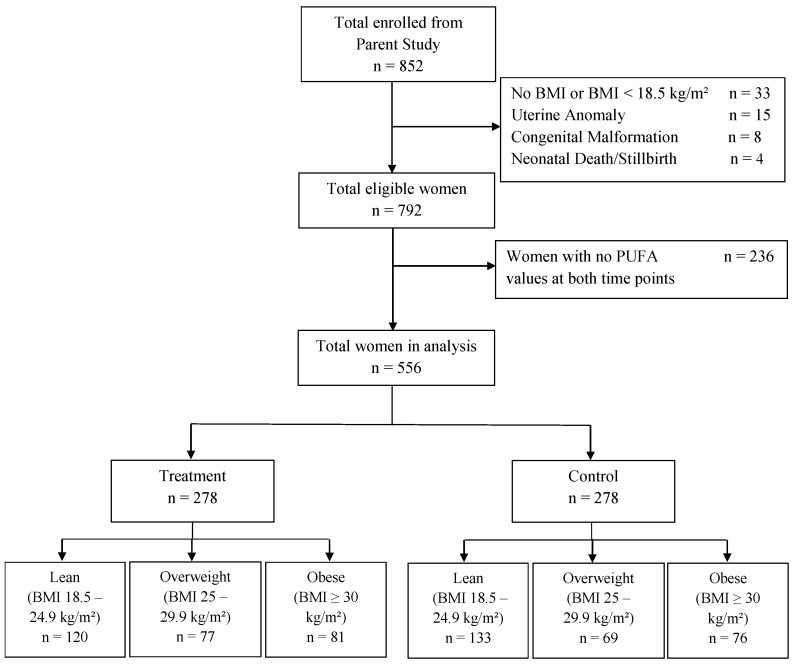
Flow diagram of the included participants. *n*: number.

This is a secondary analysis of the Maternal-Fetal Medicine Units Network randomized controlled trial (RCT) of n-3 PUFA supplementation to prevent recurrent preterm birth (ClinicalTrials.gov: NCT00135902) [28]. Participants were recruited between January 2005 and October 2006. Inclusion criteria for this RCT were a history of at least one prior singleton preterm delivery after spontaneous preterm labor or premature rupture of the membranes, and a current singleton pregnancy between 16 and 21 6/7 weeks. Detailed study procedures including exclusion criteria were described previously [28]. A total of 852 women were randomized to receive, from recruitment until delivery, either a daily supplement containing 800 mg of DHA +1200 mg of EPA (total of 2000 mg of n-3 PUFA), or placebo capsules containing a minute amount of inert mineral oil. All n-3 capsules also contained 10 international units of vitamin E as a preservative. Weekly 17-hydroxyprogesterone injections were provided for preterm birth prevention to all study participants. For this secondary analysis, we additionally excluded participants who had a uterine anomaly, a congenital malformation, or an intrauterine fetal demise/stillbirth or neonatal death. We also excluded women with a BMI <18.5 kg/m^2^ since underweight status may affect the response to n-3 supplementation via mechanisms that are different than those related to obesity. Finally, women without a recorded BMI, or who did not have PUFAs measured at any time point, were excluded as these were our exposure and outcome variables, respectively (Figure 1). All participants provided written informed consent. The institutional review board (IRB) of each clinical site approved the parent study. The current analysis was performed on the publicly released dataset of the trial under the IRB waiver by our institution (2017P000704). The dataset was released on February 2016 and is available by request at the George Washington University Biostatistics Center.

### 2.2. Exposure

Our exposure in Aim 1 was maternal pre-pregnancy BMI categorized as follows: lean (BMI 18.5 to 25 kg/m^2^), overweight (25 to 30 kg/m^2^), and obese (≥30 kg/m^2^). Research assistants calculated BMI using height measured at the first research visit and pre-pregnancy weight obtained from medical records or self-reported at the first research visit. BMI was analyzed categorically. Our exposure in Aim 2 was treatment arm (placebo and treatment groups). Here, the BMI category was considered an effect modifier.

### 2.3. Outcomes

Plasma assays. Research assistants collected blood at the enrollment visit and again between 25 and 28 weeks of gestation for plasma fatty acids analysis using methods previously described [29]. Briefly, samples were collected in ethylenediaminetetraacetic acid-tubes and were centrifuged to separate the plasma fraction. Plasma were stored at −70 °C for later analysis. Phospholipids were separated by thin-layer chromatography and were eluted with chloroform methanol in preparation for fatty acid extraction. Fatty acids were extracted using a modified procedure described by Folch et al. and were methylated with 14% boron trifluoride methanol before analysis [29]. Fatty acid methyl esters (FAME) were analyzed with the use of temperature-programmed microcapillary gas liquid chromatography with flame ionization detection (GC-FID) on a 30 mm × 0.25 mm inner diameter SP-2380 (Supelco, Bellefonte, PA, USA) column. Individual FAME were identified by comparison of retention times with known FAME standards (Nu-chek-Prep, Elysian, MN, USA). Individual PUFAs were assayed and expressed as the percent of total fatty acids by weight [29]. The concentrations of four n-3 PUFAs (α-Linolenic acid (ALA), Eicosatrienoic acid (ETE), EPA, and DHA) and four n-6 PUFAs (Linoleic acid (LA), Dihomo-gamma-linolenic acid (DGLA), AA, and Docosapentaenoic acid (DPA)) were measured in the original trial. PUFA variables that were examined in our analysis were: total n-3 PUFA (sum of ALA, ETE, EPA, and DHA), DHA+EPA, total n-6 PUFA (sum of LA, DGLA, AA, and DPA), AA, n-6/n-3 PUFA ratio (total n-6 PUFA: total n-3 PUFA), and AA/DHA+EPA ratio. We evaluated these variables because they reflected supplementation (DHA+EPA), the total load of anti-inflammatory PUFA (Total n-3 PUFA), and pro-inflammatory PUFA (Total n-6 PUFA). We also chose to evaluate fatty acids that are precursors to biologically and clinically important eicosanoids and metabolites of these eicosanoids (AA) and that have been shown to be important markers in the fetal programing of child obesity (n-6/n-3 PUFA ratio, and AA/DHA+EPA) [10,21,22,24]. 

### 2.4. Confounders 

Research assistants collected information about maternal age, education, race/ethnicity, smoking, and marital status via questionnaires and maternal interviews. They administered a validated four-item (dark meat fish, canned tuna, other fish, and shellfish) food frequency questionnaire to assess fish intake at baseline and at 25–28 weeks [30]. They had participants complete compliance logs and created an overall compliance variable of the percentage of capsules they took throughout the entire study. They also recorded gestational age at enrollment, as well as the duration of supplementation between the two assays. We chose these confounders as they related to exposure and outcome. Participants received no dietary advice as part of the study and otherwise received the usual clinical care. 

### 2.5. Statistics 

Descriptive statistics were used to characterize and compare socio-demographic variables by pre-pregnancy BMI categories. We tested for normality using the Shapiro-Wilk test. We used Kruskal-Wallis test, *χ*^2^ test, or p-trend test where appropriate for comparison of the BMI categories. For Aim 1, we investigated the associations between BMI and baseline PUFA concentrations using linear regression analyses controlling for relevant confounders. For Aim 2, in our unadjusted analyses, we used Wilcoxon signed-rank tests to determine if concentrations following supplementation were significantly different than baseline. We also used Wilcoxon rank-sum tests to further assess whether the absolute changes in concentrations following supplementation differed by treatment group within each BMI category. To control for confounding variables, we examined the effect of supplementation on PUFA concentrations in all participants using linear regression analyses adjusting for maternal sociodemographics, fish consumption, BMI as a continuous variable, and duration of supplementation. We additionally examined the effect sizes within each BMI category. To assess whether the effect sizes that were observed differed by maternal BMI category, we used interaction terms (BMI category × treatment group) in our models and reported *p* values for interaction. Since compliance in the study did not vary by maternal BMI category, this covariate was not included in our final regression models. Response to supplementation was expressed as absolute change from baseline (post concentrations–baseline concentrations) and as % total fatty acids by weight or units ratio, depending on the variable of interest. Data were analyzed using STATA Statistical Software: Release 13.1 (StataCorp, College Station, TX, USA). Statistical significance was designated to be *p* < 0.05.

## 3. Results

### 3.1. Participant Characteristics 

Descriptive characteristics of the 556 women in the study population are shown in Table 1. The median (interquartile range (IQR)) maternal age and BMI at study enrollment were 27 (23, 32) years old and 27 (22, 30) kg/m^2^. In the cohort, 46% of the women were lean (Median BMI (IQR): 22 kg/m^2^ (21, 23)), 26% were overweight (Median BMI (IQR): 27 kg/m^2^ (25, 28)), and 28% were obese (Median BMI (IQR): 34 kg/m^2^ (32, 38)). Amongst all the women, 46% were white, 37% were Black non-Hispanic, 14% were Hispanics, 3% were other, and 20% ate ≥ 2 servings of fish per week at the time of enrollment. The median (IQR) length of n-3 supplementation between baseline to post-supplementation assays was 56 (42, 63) days. Compared to lean women, obese and overweight women were more likely to have fewer years of education (*p* < 0.001), to be of Black race (*p* < 0.001), smoke (*p* = 0.08), and be divorced/separated (*p* = 0.08). Obese women (24%) were also more likely to eat ≥ 2 servings of fish per week compared to overweight (17%) and lean (16%) women (*p* = 0.006) (Table 1). There were no differences in gestational age at enrollment, length of supplementation, treatment arm allocation, or study compliance by BMI. 

### 3.2. Baseline Plasma PUFA Concentrations before n-3 Supplementation by BMI

#### 3.2.1. N-3 PUFA

Median (IQR) of total n-3 PUFA and DHA+EPA concentrations in the cohort were 4.1 (3.3, 5.1) and 3.9 (3.1, 4.7) % total fatty acids, respectively (Table 2). Plasma concentrations of total n-3 PUFA (*p* = 0.16) and DHA+EPA (*p* = 0.33) did not vary by BMI category (Table 2). 

#### 3.2.2. N-6 PUFA

Median (IQR) of total n-6 PUFA and AA concentrations in the cohort were 38.0 (33.6, 40.1) and 11.7 (9.7, 13.4) % total fatty acids, respectively (Table 2). Concentrations of total n-6 PUFA did not vary by BMI category. However, AA concentrations were higher in obese compared to lean women in the unadjusted analysis (*p* = 0.001, Table 2) and adjusted analysis (*β* = 0.96% total fatty acids; confidence interval, CI: 0.13, 1.79; Table 3). There were no differences in AA concentrations in overweight compared to lean women (*β* = −0.45%; CI: −1.29, 0.39; Table 3).

#### 3.2.3. N-6/N-3 PUFA Ratio 

The total n-6/n-3 and AA/DHA+EPA ratio (IQR) in the entire cohort were 8.9 (6.9, 10.7) and 3.0 (2.4, 3.4), respectively (Table 2). The total n-6/n-3 ratio did not differ by BMI category (*p* = 0.83). The AA/DHA+EPA ratio was higher in obese women compared to lean women in the unadjusted analysis (*p* = 0.006, Table 2) and adjusted analysis (*β* = 0.26 unit; CI: 0.05, 0.48; Table 3). There were no differences in the AA/DHA+EPA ratio in overweight compared to lean women (*β* = 0.12%; CI: −0.10, 0.35; Table 3).

### 3.3. Change in Plasma PUFA Concentrations Following n-3 PUFA Supplementation by BMI

#### 3.3.1. N-3 PUFA

There was a significant increase in total n-3 PUFA concentrations in the treatment group following n-3 supplementation (∆% total fatty acids median (IQR): 1.5% (−0.3, 5.1); *p* < 0.05) (Table 4). This rise was greater for all women in the treatment compared to the placebo group in the unadjusted (*p* < 0.000, Table 4) and adjusted analysis (*β* = 1.62% total fatty acids difference; CI: 0.03, 3.21; *p* = 0.046 Table 5). Furthermore, this effect did not differ by BMI category (Lean: *β* = 2.52%; CI: −0.16, 5.20; Overweight: *β* = 2.33%; CI: −0.16, 4.81; Obese: *β* = 0.19%; CI: −2.98, 3.22; *p* for interaction = 0.23; Table 5). 

Similarly, there was a significant increase in total DHA+EPA concentrations in the treatment group following n-3 supplementation (∆% median (IQR): 2.3% (0.0, 5.3; *p* < 0.05) (Table 4). This rise was greater for all women in the treatment compared to the placebo group in the unadjusted (*p* < 0.000, Table 4) and adjusted analysis (*β* = 3.08% total fatty acids; CI: 2.59, 3.56; *p* = 0.000; Table 5). Furthermore, the observed effects differed by BMI category (Lean: *β* = 4.03%; CI: 3.24, 4.82; Overweight: *β* = 2.14%; CI: 1.17, 3.10; Obese: *β* = 2.12%; CI: 1.32, 2.92; *p* for interaction = 0.000; Table 5), whereby obese and overweight women had an attenuated response to n-3 supplementation compared to lean women. The change that was observed following supplementation in overweight women was lower by 1.81% total fatty acids (CI: −2.99, −0.63) compared to the change that was seen in lean women. Similarly, the change that was observed following supplementation in obese women was lower by 2.04% total fatty acids (CI: −3.19, −0.90) compared to the change seen in lean women, which was equivalent to a 50% difference in the effect size between these two BMI groups.

#### 3.3.2. N-6 PUFA 

There was a significant reduction in total n-6 PUFA concentrations in the treatment group following n-3 supplementation (∆% total fatty acids median (IQR): −2.4% (−5.4, 2.1); *p* < 0.05; Table 4). This reduction was greater for women in the treatment compared to the placebo group in the unadjusted analysis (*p* = 0.02, Table 4). However, in the adjusted analysis, there was no longer a difference between the placebo and treatment groups (*β* = −0.46%; CI: −2.41, 1.49; *p* = 0.64; Table 5). Furthermore, the effects that were observed did not differ by BMI category (*p* for interaction = 0.43).

Similarly, there was a significant reduction in AA concentrations in the treatment group following n-3 supplementation (∆% median (IQR): −1.5% (−3.1, −0.3); *p* < 0.05; Table 4). This reduction was greater for women in the treatment compared to the placebo group in the unadjusted analysis (*p* = 0.001, Table 4). However, in the adjusted analysis, this difference was not significant (*β* = −0.42% total fatty acids difference; CI: −1.12, 0.27; *p* = 0.24; Table 5). Furthermore, the effects that were observed did not differ by BMI category (*p* for interaction = 0.19).

#### 3.3.3. N-6/N-3 PUFA Ratio

There was a significant reduction in the total n-6/n-3 PUFA ratio in the treatment group following n-3 supplementation (∆% median (IQR): −2.9 unit (−6.1, 0.9); *p* < 0.05; Table 4). This reduction was greater for women in the treatment compared to the placebo group in the unadjusted (*p* < 0.000, Table 4) and adjusted analysis (*β* = −2.71 units; CI: −3.70, −1.72; *p* = 0.000; Table 5). Furthermore, the change in n-6/n-3 PUFA that was seen following supplementation differed by BMI (Lean: *β* = −3.67%; CI: −5.13, −2.21; Overweight: *β* = −2.83%; CI: −5.16, −0.51; Obese: *β* = −1.55%; CI: −3.41, −0.30; *p* for interaction = 0.017; Table 5), whereby obese and overweight women had attenuated changes compared to lean women. The change that was observed following supplementation in obese women was lower by 2.30% total fatty acids (CI: −0.06, 4.67) compared to the change seen in lean women, which was equivalent to a 60% difference in the effect size between these two BMI groups.

Similarly, following n-3 supplementation, there was a significant reduction in the AA/DHA+EPA ratio as well (∆% median (IQR): −1.4 unit (−2.0, −0.4); *p* < 0.05; Table 4). This reduction was greater for women in the treatment compared to the placebo group in the unadjusted (*p* < 0.000, Table 4) and adjusted analysis (*β* = −1.13 units; CI: −1.36, −0.91; *p* = 0.000; Table 5). Furthermore, this change in the AA/DHA+EPA ratio that was seen following supplementation differed by BMI (Lean: *β* = −1.48 unit; CI: −1.78, −1.17; Overweight: *β* = −1.21; CI: −1.80, −0.63; Obese: *β* = −0.52; CI: −0.94, −0.10; *p* for interaction = 0.046; Table 5). The change that was observed following supplementation in obese women was lower by 0.94 units (CI: 0.40, 1.47) compared to the change seen in lean women, which was equivalent to a 65% difference in the effect size between these two BMI groups. 

## 4. Discussion

PUFAs play a critical role in fetal development and pregnancy health, however there is minimal data available on the role of maternal BMI in the balance of pro- and anti-inflammatory PUFAs during pregnancy. Here, we have shown that women who enter pregnancy obese have higher concentrations of the mostly inflammatory n-6 Arachidonic PUFA and an attenuated systemic response to n-3 PUFA supplementation.

Although several studies have examined the associations of maternal PUFA status with various outcomes, few studies have compared PUFA concentrations in women of different BMI classes [31,32,33]. Results by Wijendran et al. were limited by its very small sample size (*n* = 30) in which only three were obese [31]. Tomedi et al. showed in a prospective cohort study of 129 pregnant women, that those entering pregnancy obese had lower concentrations of DHA and AA. However, a comprehensive measure of other PUFAs was lacking in this cohort [32]. Consistent with our results, obese, compared to lean women in the Dutch Generation R cohort, had higher plasma n-6 PUFA [33]. However, they also had lower plasma n-3 PUFA, which was not observed in our cohort. This difference may be due to the characteristics of our cohort, which comprised of women with a history of preterm birth receiving 17-hydroxyprogesterone, which may modulate n-3 PUFA synthesis [34]. Additionally, obese, compared to lean women in our cohort, had a higher consumption of fish, which may not apply to the Dutch population. In our study, obese women at baseline had AA concentrations that were 10% higher compared to lean women. It is unclear whether this represents a clinically significant change as no studies have examined the effects of a dose-response increase in AA on health outcomes. However, these results are similar to the trend observed in the Generation R study, where obese women had AA concentrations that were less than 0.5 standard deviation lower compared to lean women, suggesting a small difference. Together with our study, results from previous studies support associations of maternal obesity with an inflammatory PUFA profile. Future studies should characterize n-3 PUFA and AA-derived lipid mediator profiles that are associated with maternal obesity to assess the degree of obesity-induced inflammation, and determine whether these profiles contribute to the developmental programming of adverse outcomes that disproportionally affect children of obese women.

Dietary patterns in obese individuals likely contribute to this unfavorable PUFA profile, which in turn further exacerbates the intrinsic low-grade chronic inflammation of obesity. Dietary shift over the years to a Western diet has caused a drastic increase in the dietary ratio of n-6/n-3 PUFA from about 2:1 in the Paleolithic diet to about 15–20:1 [35]. Obesity is associated with the consumption of a Western diet, which is deficient in n-3 PUFA and, specifically, in DHA. Animal studies and human trials also suggest that higher BMI and n-6/n-3 PUFA ratio are associated with the activation of distinct placental inflammatory pathways and dysregulation of genes that are pertinent to cytokine production and lipid metabolism [36,37,38,39]. Furthermore, even breastmilk of obese mothers is characterized by higher n-6/n-3 PUFA ratio, mirroring systemic levels, and this profile, in i*n-vitro* studies, is associated with higher inflammatory cytokines and increased expression of genes that are involved in lipogenesis [40,41,42]. Given the potential repercussions on future generations, further studies are urgently needed to examine the role of dietary PUFAs and dietary inflammation on placental insufficiency and lipotoxicity in obese pregnancies. 

We described an attenuated systemic response to n-3 PUFA supplementation in overweight and obese pregnant women, which has previously been observed only in small, non-pregnant cohorts. Christian et al. examined the effects of BMI on PUFA accumulation in 64 youth in a RCT of n-3 PUFA supplementation and demonstrated similarly that a higher BMI category predicted a less robust increase in n-3 PUFAs [43]. Another study of 48 women with increased breast cancer risk supplemented with n-3 PUFAs showed reduced changes in serum and breast adipose tissue levels in women with higher BMI [44]. Our results uniquely suggest that n-3 supplementation in obese women in pregnancy may not achieve similar concentrations as in lean women. This raises a question for future studies of whether obese women need higher n-3 PUFA dosing to achieve similar concentrations.

The unique metabolic milieu of obese pregnancies may contribute to these attenuated changes that are seen following n-3 supplementation. Adipocyte fatty acid-binding proteins are adipokines that are increased in obese individuals and which bind to exogenous or endogenous fatty acids [45]. Furthermore, obesity is associated with increased oxidative stress which might induce lipid peroxidation and free-radical mediated DNA damage of genes involved in PUFA metabolism [46]. Finally, studies suggest that circulating sex hormones, which are often disturbed in obesity, may play a role in the availability and metabolism of circulating PUFA [34]. These potential mechanisms suggest that obesity-specific factors may play an inhibitory role in the metabolism and bioavailabity of n-3 PUFAs, which might lead to lower availability of n-3 PUFAs for obese women and their fetuses throughout pregnancy. Supplementation trials with pharmacokinetics and pharmacodynamics studies aimed at understanding factors in obesity that may modulate response to n-3 supplementation are needed. 

Our study is not without limitations, mainly due to the inclusion and exclusion criteria of the parent trial. This is a selective cohort of women with a history of preterm birth, therefore, the results may not apply to other populations of pregnant women. Furthermore, no detailed dietary information was collected as part of the original trial; therefore, we were unable to adjust for types of fats and energy intake during the supplementation period. This limitation could potentially attenuate the strength of our findings. Studies examining the clinical determinants of blood levels of EPA and DHA consistently indicate that diet (total dietary fat) is the main predictor, although other factors such as waist girth, triglyceride levels, and physical activity are commonly identified as predictors as well [47,48,49]. Therefore, future studies examining relationships between BMI and response to omega-3 supplementation should explore these confounders as well. Erythrocyte PUFAs are the ideal biomarkers for habitual dietary fat intake as they reflect a longer period of intake; however, we chose to use plasma PUFA measurements as they would more accurately reflect the shorter time frame of dietary intake between the two assays. Another methodological limitation was in the analysis of the fatty acids by GC-FID, which expresses fatty acids as % of total fatty acids by weight. As a result, the relative levels reported are affected by the levels of other fatty acids that are present. Expressing fatty acids as absolute amounts (e.g mg/dL) would enable comparisons between studies and the comparison of changes in concentration over time within the same group. However, a recent systematic review of the blood fatty acids data of healthy adults worldwide revealed that 78% of the data were reported as weight percentage overall, with higher reporting percentages in the erythrocyte (91%), whole blood (92%), and plasma PL (83%) blood fractions [50]. Another limitation may have been the presence of small amounts of vitamin E in the omega-3 capsules, which could theoretically interfere with PUFA levels. Vitamin E is an antioxidant which stabilizes DHA-rich cell membranes from oxidative stress and lipid peroxidation. However, we believe that vitamin E was present in too small amounts to have a significant systemic effect. Additionally, treatment arm allocation did not differ by BMI group. Therefore, the effects we are seeing are unlikely due to the presence of vitamin E in the capsules. Finally, although there is a possibility that a larger volume of distribution associated with higher BMI may lead to hemodilution of PUFAs, it is important to note that BMI did not attenuate all PUFAs following supplementation. Only specific PUFAs that are important modulators of inflammation were affected, suggesting that the changes that were observed were not due to hemodilution alone. Furthermore, studies have also suggested that associations of obesity with lower n-3 contents may be mediated by increased adiposity, oxidative stress (higher susceptibility to lipid peroxidation and other pro-oxidant events), and inflammation, and not uniquely by increased volume of distribution [47,48,49].

This study presents several unique strengths. We leveraged data from an existing RCT for this analysis, thus, there was less residual confounding than in observational studies. We also adjusted for characteristics that may have been unbalanced among the BMI groups. Another strength is the availability of the placebo group; we showed that the differences that were observed between the BMI categories were not due to pregnancy alone or the placebo effect. Finally, the population characteristics in this cohort are representative of the United States: racially diverse, with overall low fish consumption, and a prevalence rate in obesity and overweight that mirrors the US population, making our results generalizable.

## 5. Conclusions

In summary, our findings suggest that obese women have an attenuated response to n-3 PUFA supplementation compared to lean women. Given the burden of maternal obesity on maternal and infant health care outcomes and the crucial role of n-3 PUFA in fetal and postnatal development, future studies should seek to understand optimal n-3 PUFA intake and dosing for obese and overweight women. Obese women are at higher risk for adverse pregnancy and offspring outcomes associated with low n-3 PUFA status; therefore, future trials should also explore whether optimizing n-3 PUFA intake through weight- or BMI-based diet and supplementation strategies can improve health outcomes for these high-risk mother-infant dyads.

## Figures and Tables

**Table 1:** Baseline characteristics of participants, overall and by maternal BMI category.

Characteristics	Total *n* = 556	Pre-Pregnancy BMI (kg/m^2^) Category			*p*-Value ^a^
		Lean *n* = 253 (46%)	Overweight *n* = 146 (26%)	Obese *n* = 157 (28%)	
Age at enrollment (years)	27 (23, 32)	27 (24, 32)	28 (23, 33)	27 (23, 32)	0.79
BMI (kg/m^2^)	26.5 (22, 30)	22 (21, 23)	27 (25, 28)	34 (32, 38)	<0.001 ^b,c,d^
Gestational age at randomization (days)	136 (125, 146)	135 (124, 146)	138 (126, 147)	135 (125, 145)	0.43
Education (years)	13 (12, 16)	14 (12, 16)	12 (11, 15)	12 (12, 14)	<0.001 ^c,d^
Race/Ethnicity ^e^					
White	254 (46)	142 (56)	57 (39)	55 (35)	<0.001
Black, non-Hispanic	204 (37)	63 (25)	58 (40)	83 (53)	
Hispanic	81 (14)	36 (14)	28 (19)	17 (11)	
Other	16 (3)	11 (4)	3 (2)	2 (1)	
Smoking in pregnancy (Yes, %)	89 (16)	32 (13)	24 (16)	33 (21)	0.08
Marital Status					
Married/Living with Partner	371 (67)	183 (72)	95 (65)	93 (59)	0.08
Divorced/Widowed/Separated	29 (5)	9 (4)	9 (6)	11 (7)	
Never Married	156 (28)	61 (24)	42 (29)	53 (34)	
Length of supplementation (days)	56 (42, 63)	55 (41, 64)	56 (40, 63)	55 (42, 63)	0.84
Fish intake (servings/week)					
<1	269 (48)	136 (54)	71 (49)	62 (40)	0.006 ^f^
1	183 (33)	76 (30)	50 (34)	57 (36)	
2	41 (8)	13 (5)	10 (7)	18 (11)	
≥3	63 (11)	28 (11)	15 (10)	20 (13)	
Treatment Arm					
Treatment	278 (50)	120 (47)	77 (53)	81 (52)	0.53
Placebo	278 (50)	133 (53)	69 (47)	76 (48)	
Study Compliance (%)	92 (81, 99)	93 (83, 99)	93 (82, 99)	89 (78, 99)	0.15

Data are median (interquartile range), or *n* (%). BMI, Body Mass Index. ^a^ *p*: Non-parametric Kruskal-Wallis test for comparison by BMI categories for continuous variables; chi-squared test for categorical variables; *p*-trend test for ordered categorical variables (fish intake); ^b,c,d^ Statistical significance (*p* < 0.001) for comparison of obese vs. overweight ^b^, obese vs. lean ^c^, overweight vs. lean ^d^, in Kruskal-Wallis pairwise analysis with Bonferroni correction; ^e^ One participant with unknown race/ethnicity; ^f^ Significant linear trend in fish intake across BMI categories.

**Table 2:** Baseline plasma PUFA concentrations, overall and by maternal BMI category.

	*n*	Total	Pre-Pregnancy BMI (kg/m^2^) Category			*p*-Value ^a^
			Lean	Overweight	Obese	
n-3 PUFA						
Total n-3 PUFA	532	4.1 (3.3, 5.1)	4.1 (3.4, 5.2)	3.9 (3.1, 5.0)	4.1 (3.4, 5.0)	0.16
DHA + EPA	532	3.9 (3.1, 4.7)	3.9 (3.1, 4.7)	3.8 (2.7, 4.6)	4.0 (3.2, 4.6)	0.33
n-6 PUFA						
Total n-6 PUFA	532	38.0 (33.6, 40.1)	37.9 (33.2, 40.0)	38.3 (34.1, 40.2)	37.9 (33.7, 40.1)	0.73
AA	532	11.7 (9.7, 13.4)	11.5 (9.3, 13.0)	11.5 (9.4, 13.0)	12.6 (10.6, 14.6)	0.001 ^b,c^
n-6/n-3 PUFA ratio						
Total n-6/n-3 PUFA	477	8.9 (6.9, 10.7)	9.0 (6.7, 10.6)	8.8 (6.7, 10.9)	8.8 (7.1, 10.5)	0.83
AA/DHA + EPA	461	3.0 (2.4, 3.4)	2.9 (2.3, 3.3)	3.0 (2.3, 3.5)	3.1 (2.7, 3.6)	0.006 ^c^

Data are median (interquartile range) in percentage of total fatty acids by weight. BMI, Body Mass Index; PUFA, Polyunsaturated Fatty Acids; DHA, Docosahexaenoic acid; EPA, Eicosapentaenoic acid; AA, Arachidonic acid. ^a^ Non-parametric Kruskal-Wallis test for comparison by BMI categories; ^b,c^ Statistical significance (*p* < 0.01) for comparison of obese vs. overweight ^b^, obese vs. lean ^c^ in Kruskal-Wallis pairwise analysis with Bonferroni correction.

**Table 3:** Estimated difference (*β*) and 95% confidence interval in baseline PUFA concentrations between BMI category.

	*n*	Model 0	Model 1	Model 2
Total n-3 PUFA	531			
Lean		Ref	Ref	Ref
Overweight		−1.97 (−3.51, −0.43) ^a^	−1.80 (−3.39, −0.21) ^a^	−1.80 (−3.39, −0.21) ^a^
Obese		−0.96 (−2.46, 0.55)	−0.58 (−2.15, 1.00)	−0.57 (−2.15, 1.00)
DHA+EPA	531			
Lean		Ref	Ref	Ref
Overweight		−0.25 (−0.61, 0.11)	−0.25 (−0.61, 0.11)	−0.24 (−0.60, 0.11)
Obese		−0.11 (−0.24, 0.46)	0.16 (−0.20, 0.52)	0.13 (−0.22, 0.49)
Total n-6 PUFA	531			
Lean		Ref	Ref	Ref
Overweight		0.68 (−1.14, 2.50)	0.45 (−1.42, 2.31)	0.47 (−1.39, 2.34)
Obese		0.48 (−1.29, 2.26)	0.21 (−1.64, 2.06)	0.15 (−1.70, 1.99)
AA	531			
Lean		Ref	Ref	Ref
Overweight		−0.24 (−1.05, 0.57)	−0.47 (−1.31, 0.37)	−0.45 (−1.29, 0.39)
Obese		1.30 (0.51, 2.10) ^a^	1.00 (0.17, 1.83) ^a^	0.96 (0.13, 1.79) ^a^
Total n-6/n-3 PUFA	476			
Lean		Ref	Ref	Ref
Overweight		0.24 (−0.62, 1.10)	0.25 (−0.65, 1.14)	0.24 (−0.65, 1.14)
Obese		0.21 (−0.61, 1.03)	0.26 (−0.61, 1.13)	0.27 (−0.60, 1.15)
AA/DHA+EPA	460			
Lean		Ref	Ref	Ref
Overweight		0.19 (−0.04, 0.41)	0.12 (−0.10, 0.35)	0.12 (−0.10, 0.35)
Obese		0.35 (0.14, 0.56) ^a^	0.26 (0.04, 0.47) ^a^	0.26 (0.05, 0.48) ^a^

Beta represents differences (in percentage total fatty acids by weight or units ratio) in baseline plasma PUFA concentrations between obese and overweight compared to lean category. Ref, reference group; BMI, Body Mass Index; PUFA, Polyunsaturated Fatty Acids; DHA, Docosahexaenoic acid; EPA, Eicosapentaenoic acid; AA, Arachidonic acid. ^a^ Result indicates significant difference compared to lean group; Model 0 is the unadjusted model; Model 1 adjusts for maternal age, race, smoking, education, marital status, gestational age at randomization; Model 2 adjusts for all covariates in Model 1 and fish intake at baseline.

**Table 4:** Absolute change in PUFA concentrations following n-3 supplementation by treatment category, stratified by BMI.

	*n*	Treatment Group		*p*-Value ^a^
		Placebo	Treatment	
Total n-3 PUFA				
All	472	−0.2 (−1.2, 0.6) ^b^	1.5 (−0.3, 5.1) ^b^	<0.000
Lean		−0.02 (−1.6, 0.6)	3.4 (−0.2, 6.6) ^b^	<0.000
Overweight		−0.2 (−1.6, 0.6)	1.5 (0.0, 4.2) ^b^	<0.000
Obese		−0.6 (−1.5, 0.3) ^b^	0.4 (−0.8, 2.5)	0.002
DHA + EPA				
All	472	−0.2 (−0.9, 0.4) ^b^	2.3 (0.0, 5.3) ^b^	<0.000
Lean		−0.1 (−0.9, 0.5)	4.5 (0.7, 6.7) ^b^	<0.000
Overweight		−0.2 (−0.6, 0.4)	1.9 (0.0, 4.2) ^b^	<0.000
Obese		−0.5 (−1.2, 0.0) ^b^	1.9 (0.0, 4.2) ^b^	<0.000
Total n-6 PUFA				
All	472	−0.3 (−3.5, 2.7)	−2.4 (−5.4, 2.1) ^b^	0.02
Lean		0.6 (−3.4, 2.2)	−3.7 (−6.5, 1.6) ^b^	0.01
Overweight		0.1 (−4.6, 3.3)	−2.0 (−5.2, 2.7)	0.32
Obese		−0.2 (−3.4, 2.2)	−0.5 (−4.2, 2.2)	0.65
AA				
All	472	−0.9 (−2.3, 0.4) ^b^	−1.5 (−3.1, −0.3) ^b^	0.001
Lean		−0.7(−1.8, 0.4) ^b^	−2.0 (−3.5, −0.8) ^b^	<0.000
Overweight		−1.1 (−2.0, 0.4) ^b^	−1.5 (−3.0, 0.0) ^b^	<0.000
Obese		−1.0 (−3.1, 0.0) ^b^	−1.0 (−2.2, −0.1) ^b^	0.54
Total n-6/n-3				
All	396	0.3 (−1.2, 1.6)	−2.9 (−6.1, 0.9) ^b^	<0.000
Lean		−0.1 (−1.8, 1.4)	−4.3 (−7.1, −0.1) ^b^	<0.000
Overweight		0.5 (−0.6, 1.6)	−2.8 (−5.3, 1.2) ^b^	<0.000
Obese		1.0 (−0.9, 2.0)	−1.1 (−3.8, 1.5) ^b^	0.016
AA/DHA + EPA				
All	384	−0.1 (−0.4, −0.2)	−1.4 (−2.0, −0.4) ^b^	<0.000
Lean		−0.1 (−0.3, 0.1)	−1.6 (−2.1, −0.8) ^b^	<0.000
Overweight		0.0 (−0.4, 0.3)	−1.4 (−2.2, −0.5) ^b^	<0.000
Obese		−0.1 (−0.6, 0.2)	−0.5 (−1.5, −0.1) ^b^	0.002

Data are median (interquartile range) in percentage of total fatty acids by weight or units ratio. BMI, Body Mass Index; PUFA, Polyunsaturated Fatty Acids; DHA, Docosahexaenoic acid; EPA, Eicosapentaenoic acid; AA, Arachidonic acid. ^a^ *p*-values for comparison between placebo and control groups by Wilcoxon rank-sum test; ^b^ *p* < 0.05: Statistical significant difference comparing baseline vs. post-supplementation levels by Wilcoxon signed-rank.

**Table 5:** Adjusted estimated difference (*β*) in absolute change in PUFA concentrations in treatment compared to placebo group following n-3 supplementation, overall, and stratified by BMI.

	*n*	Overall	*p* ^a^	Pre-Pregnancy BMI Category			*p* for Interaction
				Lean	Overweight	Obese	
n-3 PUFA							
Total n-3 PUFA	471	1.62 (0.03, 3.21)	0.046	2.52 (−0.16, 5.20)	2.33 (−0.16, 4.81)	0.19 (−2.98, 3.22)	0.230
DHA + EPA	471	3.08 (2.59, 3.56)	0.000	4.03 (3.24, 4.82) ^b^	2.14 (1.17, 3.10) ^b^	2.12 (1.32, 2.92) ^b^	0.000
n-6 PUFA							
Total n-6 PUFA	471	−0.46 (−2.41, 1.49)	0.640	−1.70 (−4.59, 1.19)	1.17 (−5.69, 3.36)	0.21 (−3.17, 3.58)	0.430
AA	471	−0.42 (−1.12, 0.27)	0.240	−1.13 (−2.16, −0.11) ^b^	−0.67 (−2.08, 0.74)	0.53 (−0.87, 1.93)	0.190
n-6/n-3 PUFA ratio							
Total n-6/n-3 PUFA	395	−2.71 (−3.70, −1.72)	0.000	−3.67 (−5.13, −2.21) ^b^	−2.83 (−5.16, −0.51) ^b^	−1.55 (−3.41, −0.30) ^b^	0.017
AA/ DHA + EPA	383	−1.13 (−1.36, −0.91)	0.000	−1.48 (−1.78, −1.17) ^b^	−1.21 (−1.80, −0.63) ^b^	−0.52 (−0.94, −0.10) ^b^	0.046

Beta (CI) represents adjusted estimated differences (in percentage total fatty acids or units ratio) in the change of plasma PUFA concentrations in the treatment group compare to the placebo group (reference), overall for all participants, and stratified by BMI with 95% Confidence Interval. Estimates are adjusted for maternal age, race, smoking, education, marital status, fish intake, BMI and length of supplementation; BMI, Body Mass Index; PUFA, Polyunsaturated Fatty Acids; DHA, Docosahexaenoic acid; EPA, Eicosapentaenoic acid; AA, Arachidonic acid. ^a^ *p*-values for effect estimates in overall cohort; ^b^ Result indicates significant difference in the effect size in treatment group compared to placebo group.
